# Trends of Dispensed Opioids Prescribed by Australian Dentists: 2013–2022

**DOI:** 10.1111/cdoe.70022

**Published:** 2025-10-07

**Authors:** Leanne Teoh, Marietta Taylor, Erin Kelty, Frank M. Sanfilippo, Mathew Lim, Michael McCullough, Christopher Etherton‐Beer, Alex Park, David Preen, Amy Page

**Affiliations:** ^1^ Melbourne Dental School University of Melbourne Victoria Australia; ^2^ School of Population and Global Health University of Western Australia Perth Australia; ^3^ Centre for Optimisation of Medicines University of Western Australia Perth Australia; ^4^ WA Centre for Health & Ageing University of Western Australia Perth Australia

**Keywords:** Australian dental prescribing, dental prescribing, opioid misuse, opioid stewardship, opioids, prescribing trends

## Abstract

**Background:**

In Australia, the prescribing of opioid medicines by dentists has increased in recent years, despite opioids not being first‐line treatment for dental pain. The aim of this longitudinal study was to examine the dispensing of opioids prescribed by dentists in Australia during 2013–2022.

**Method:**

A nationally representative 10% sample of patients identified from the Australian Pharmaceutical Benefits Scheme dispensing data from 2013 to 2022 was used. Three outcomes were assessed: (1) incidence of dispensing of all dental prescriptions; (2) incidence of dispensing of opioids prescribed by dentists; (3) average number of tablets/capsules of dental opioid supply. Outcomes pertaining to opioid use were examined overall, and by year, age and sex.

**Results:**

From 2013 to 2022, 998 774 dental prescriptions (of any kind) were dispensed to 470 118 patients. The mean annual incidence rate for dispensing any dental medication was 48.4 (95% CI: 48.3–48.5)/1000 person‐years. Opioids accounted for 183 303 prescriptions (18.4%), with a mean annual incidence rate of 11.0 (95% CI: 11.0–11.1)/1000 person‐years. The majority of patients (99.1%) were dispensed ≤ 4 opioid prescriptions across the 10‐year period, with 0.9% of patients (*n* = 1312) receiving between 5 and 149 dispensed opioids. Over the study period, the average annual incidence of dispensed dental opioids increased by 4.4% (95% CI: 1.0–8.2). A reduction in the mean quantity of opioid pills dispensed was observed over time. Dental opioids were dispensed to 2727 children and adolescents.

**Conclusion:**

The incidence of dispensing of dental opioids in Australia increased by an average of 4.4% per year over a decade. While there was a reduction in opioid quantities dispensed, dispensing of opioids for children occurred, and a small number of patients were dispensed excessive quantities of dental opioids. Evidence‐based tailored opioid stewardship interventions need to include dentists, and dentists should be provided access to drug monitoring programmes to enable more informed prescribing decisions.

## 1 Introduction

Dental pain is a common presentation managed by dentists, where opioids are frequently prescribed [1, 2]. Dentists are the second highest prescribers of opioids in the United States (US), although prescribing rates have decreased in recent years [3, 4]. However, an Australian study has shown that dental opioid dispensing has increased nationally [5]. Australian dental opioid dispensing is relatively high compared with other countries, with Australian dental opioids being dispensed 21 times more often compared to English dentists by population [5].

Australian guidelines recommend that opioids are not first‐line treatment for dental pain, with nonsteroidal anti‐inflammatory drugs (NSAIDs) being recommended initially [6]. Inconsistent with guidance, research suggests that 16%–27% of Australian dentists would prescribe an opioid, acetaminophen (paracetamol) or an opioid combined with acetaminophen as the first choice for dental pain, without a NSAID, for invasive dental procedures such as surgical tooth extraction [2].

Opioids have limited effectiveness for dental pain [7]. A systematic review demonstrated that the effectiveness of commonly prescribed doses of oxycodone and codeine in dentistry are similar to placebo [7]. Additionally, opioids carry risks of dependence and abuse, as well as serious opioid‐related adverse effects, including those that require hospitalisation and emergency department attendance [8]. Thus, understanding dental opioid prescribing patterns and choices is important. While there have been previous studies of dispensed opioids prescribed by dentists using aggregated datasets [5, 9], there have been no studies assessing individual prescription patterns linked to population demographics in Australia. Establishing current information on dispensings of opioids prescribed by dentists can assist with developing targeted stewardship interventions to improve prescribing. Thus, the aim of this study was to assess dispensing patterns of opioids prescribed by dentists in the Australian population from 2013 to 2022.

## 2 Method

### 2.1 Study Design

This was a longitudinal, retrospective study that utilised a nationally representative 10% sample of Australian patients who had records in the Pharmaceutical Benefits Scheme (PBS) database. The PBS is part of Australia's National Medicines Policy, where the Australian Government subsidises the cost of most medicines for most medical conditions through this scheme. This enables the provision of affordable and necessary medicines for Australians. Data were extracted for all PBS records of these patients over a 10‐year period from 2013 to 2022. This study followed the Strengthening the Reporting of Observational Studies in Epidemiology (STROBE) reporting guideline [10] (Table S1). The study protocol was reviewed and approved by the University of Western Australia Human Research Ethics Committee (2022/ET000371).

### 2.2 Data Source

We obtained data from a random 10% sample of patients who had dispensings in the PBS database from the Australian Government Department of Health that includes government‐subsidised prescriptions dispensed in community pharmacies, and public and private hospitals. Prescribers are identified in the dispensing record with a unique identification code and the prescriber type. We identified overall and opioid prescriptions where the prescriber type was a ‘dentist’ [11].

Dentists are eligible to prescribe a limited range of PBS medications [12]. Given the acute and limiting nature of most dental pathology managed by dentists, they are not eligible to provide repeat prescriptions. Oral and maxillofacial surgeons may hold registration as both a medical and dental practitioner, so any prescriptions provided using their medical identification code would not be identified as dental prescriptions. In this dataset, PBS prescriptions were provided by 18 895 unique registered dentists across the 10‐year period. The Dental Board of Australia recorded a total of 19 407 practising dentists in 2022 [13]. Hence, the dataset captured the majority of all practising dentists (97%).

We used the Anatomical Therapeutic Chemical (ATC) codes for opioid medicines (NO2) [14], and those were listed under the dental PBS. These opioids included codeine, codeine with acetaminophen, hydromorphone, morphine, oxycodone and tramadol.

Variables included sex, date of supply, year of birth, prescriber type and year of death. Age at date of supply was calculated by subtracting the year of birth from the date of supply. Participants with missing covariate data were excluded from the analysis.

### 2.3 Outcomes

Three outcomes were assessed: (1) incidence of all dental dispensing; (2) incidence of dental opioid dispensing; (3) average number of tablets/capsules of dental opioid supply. Outcomes pertaining to opioid use were examined overall, and by year, age and sex.

### 2.4 Statistical Analysis

Characteristics of those who received a dentist prescription were summarised with means and standard deviations, confidence intervals (CI) and proportions and rates were compared using incidence rate ratios.

The annual incidence was determined by dividing the total number of events (e.g., supply of a dentist PBS medication) by the number of individuals who received a PBS prescription, and is presented as the number of dispensings per 1000 person‐years, with 95% CIs. Logistic regression was used to assess the effects of covariates on the odds of receiving a dentist opioid prescription. Temporal trends were examined by join‐point regression using Join‐point Regression Program (version 5.1.0, April 2024). Repeat events (multiple dispensings across the observation period) were included in the analysis. Statistical analyses were undertaken using Stata SE16.0; two‐tailed *p*‐values were generated, and alpha was 0.05.

## 3 Results

### 3.1 Total Number and Incidence of Dispensings of Dental Prescriptions

From 2013 to 2022, 998 774 dental prescriptions were dispensed to 470 118 patients overall. The incidence rate for any dental prescription dispensed during the observation period was 48.4 (95% CI: 48.3–48.5) prescriptions per 1000 person‐years (Table 1), and the rate did not differ significantly across the observation period (Figure S1).

**TABLE 1:** Patients dispensed any medicine and opioid medicines by a dentist in Australia between 2013 and 2022.

	All medications		Opioids	
	*N* (%)	IR (95% CI)	*N* (%)	IR (95% CI)
**All dispensings**	998 774	48.4 (48.3, 48.5)	183 303 (18.4%)	11.0 (11.0, 11.1)
**Sex**				
Female	530 319 (53.1%)	48.7 (48.6, 48.9)	91 104 (49.7%)	10.1 (10.1, 10.2)
Male	468 455 (46.9%)	48.2 (48.1, 48.4)	92 199 (50.3%)	12.1 (12.1, 12.1)
**Patient Age**				
Age (years), mean ± SD	47.9 ± 19.7		42.9 ± 16.5	
0–19	61 231 (6.1%)	16.7 (16.2, 17.2)	9339 (5.1%)	3.1 (2.9, 3.3)
20–39	280 217 (28.1%)	51.1 (50.3, 51.7)	73 908 (40.3%)	18.1 (17.6, 18.5)
40–59	345 493 (34.6%)	59.8 (59.1, 60.5)	66 761 (36.4%)	14.5 (14.1, 14.8)
60–79	273 021 (27.4%)	60.6 (59.8, 61.4)	30 819 (16.8%)	7.8 (7.6, 8.2)
80+	38 444 (3.9%)	35.0 (33.9, 36.2)	2461 (1.3%)	2.4 (2.1, 2.7)

*Note:* IR: Mean annual incidence rate per 1000 person‐years. Opioid medications: ATC codes N02AA, N02AA01, N02AA03, N02AA05, N02AJ06, N02AX02.

### 3.2 Total Number of Dispensings of Dental Opioid Prescriptions

During the observation period, the total number of opioids dispensed from dental prescriptions was 183 303, representing 18.4% of all dental prescriptions dispensed. The mean age of patients who received opioids from dental prescriptions was 42.9 years (SD: 16.5) and 50.3% were male (Table 1).

The combination product codeine 30 mg/acetaminophen 500 mg was the most commonly dispensed opioid, representing 95.4% of all dispensed dental opioid prescriptions (*n* = 174 781), followed by oxycodone (3.5%, *n* = 6257) and tramadol (1.2%, *n* = 2131). Overall, the dispensing of codeine (sole ingredient), morphine and hydromorphone from dental prescriptions was very small (Table S3).

Of individuals who were dispensed a dental opioid prescription, 80.9% (*n* = 114 844) received a single item; that is, they were supplied with a single opioid medication on a single occasion during the observation period. While 18.2% (*n* = 25 792) of patients received between two and four opioids from dental prescriptions (separate dispensings on separate dates), 0.9% (*n* = 1312) were dispensed ≥ 5 opioid prescriptions (maximum was 149 prescriptions) across the 10‐year period. There were 590 episodes where a patient was dispensed two or three different opioids from a dentist on the same day. These multiple dispensings were for a combination of codeine with acetaminophen, oxycodone and tramadol.

### 3.3 Incidence of Dispensings of Dental Opioid Prescriptions

The mean annual incidence rate for the dispensing of a dental opioid prescription was 11.0 (95% CI: 11.0–11.1) per 1000 person‐years (Table 1). Between 2013 and 2022, the annual incidence rate for dental opioid prescriptions increased from 9.9 to 12.3 per 1000 person‐years (Table S2), representing an Annual Average Percent Change (AAPC) of 4.4% (95% CI: 1.0–8.2) (Figure S1). Adjusting for age and sex, this effect persisted, and the odds of being supplied an opioid from a dentist prescription increased by an average of 6.0% each year (OR:1.06, 95% CI: 1.06–1.07) (Table 2).

**TABLE 2:** The crude and adjusted association between sex, year of dispensing and age on the dispensing of opioid medicines by dentists.

	Univariable		Multivariable	
	OR (95% CI)	*p*	OR (95% CI)	*p*
All opioids				
Sex^+^	1.18 (1.16, 1.20)	< 0.001	1.21 (1.19, 1.23)	< 0.001
Year of dispensing*	1.05 (1.05, 1.06)	< 0.001	1.06 (1.06, 1.07)	< 0.001
Age (years)				
*0–19*	0.75 (0.69, 0.82)	< 0.001	0.76 (0.70, 0.82)	< 0.001
*20–39*	1.50 (1.47, 1.52)	< 0.001	1.50 (1.48, 1.53)	< 0.001
*40–59*	1.00 (Ref)		1.00 (Ref)	
*60–79*	0.53 (0.53, 0.54)	< 0.001	0.52 (0.51, 0.53)	< 0.001
*80+*	0.29 (0.26, 0.32)	< 0.001	0.28 (0.25, 0.31)	< 0.001

^+^ Males compared to females.

* Change in risk for every unit increase in year.

**FIGURE 1:**
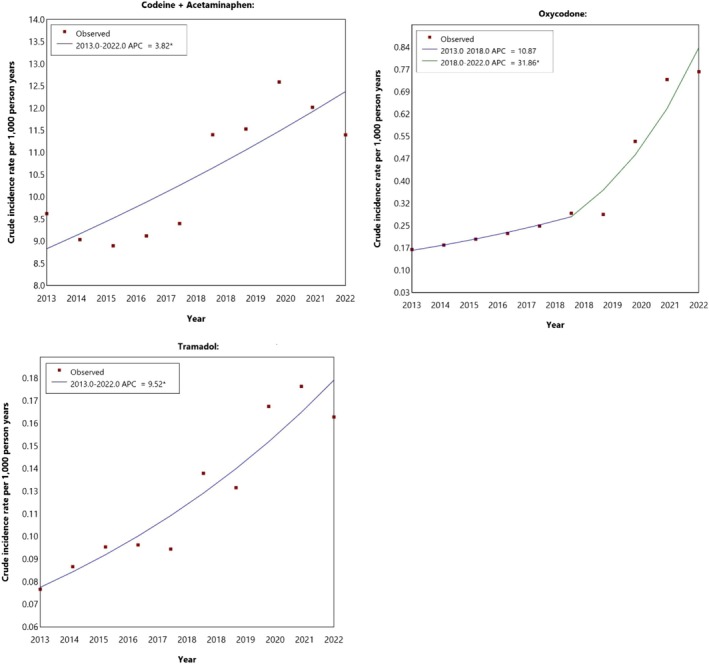
Temporal trends in annual dental opioid dispensing incidence rates, by drug type, in Australia between 2013 and 2022. *Indicates that the Annual percent change (APC) is significantly different from zero at the alpha = 0.05 level.

Between 2013 and 2022, the number of individuals who were dispensed codeine 30 mg/acetaminophen 500 mg from a dentist prescription increased from 9.6 to 11.4 per 1000 person‐years (AAPC:3.8%, 95% CI: 0.4–7.7) (Figure 1).

The incidence of oxycodone dentist prescriptions increased from 0.17 to 0.29 per 1000 person years between 2013 and 2018. This change was not statistically significant. Between 2018 and 2022, the supply of oxycodone from dental prescriptions almost tripled from 0.29 to 0.79 dispensings per 1000 person‐years. This represented an AAPC of 31.9% (95% CI: 23.4–50.0) The incidence of tramadol supply from dentist prescriptions increased from 0.08 in 2013 to 0.16 in 2022, representing an AAPC of 9.5% (95% CI: 6.0–13.9) (Figure 1), albeit the absolute rates were low.

### 3.4 Association of Dispensings of Dental Opioid Prescriptions by Age and Sex

The incidence of opioid dispensing from dental prescriptions was the highest for those aged 20–39 years, who were supplied 18.1 opioids per 1000 person‐years (95% CI: 17.6–18.5). The incidence was lowest in those aged 80 years or more, who were dispensed 2.4 opioids per 1000 person‐years (95% CI: 2.1–2.7) (Table 1).

The incidence of opioid dispensing from dental prescriptions was higher in men (IR: 12.1, 95% CI: 12.1–12.1) than in women (IR:10.1, 95% CI: 10.1–10.2) (Incidence rate ratio: 1.19, 95% CI: 1.18–1.20), *p* < 0.001) (Table 1). Adjusting for age and year, men were 21% more likely to be dispensed an opioid prescribed by a dentist than women (aOR: 1.21, 95% CI: 1.19–1.23) (Table 2).

Opioid medications comprised 7.9% (*n* = 2727) of dentist prescriptions dispensed to children and adolescents. The majority were for codeine 30 mg/acetaminophen 500 mg (*n* = 2555, 7.4%), of which 37 dispensings were to children ≤ 5 years (0.1%). Children aged 6–11 years, and 12–17 years, received 114 (0.3%) and 2404 (7.0%) prescriptions for codeine 30 mg/acetaminophen 500 mg respectively. One hundred and twenty‐one prescriptions of oxycodone were dispensed to adolescents aged 12–17 years (0.3%).

### 3.5 Average Number of Tablets/Capsules of Opioid Supply

**FIGURE 2:**
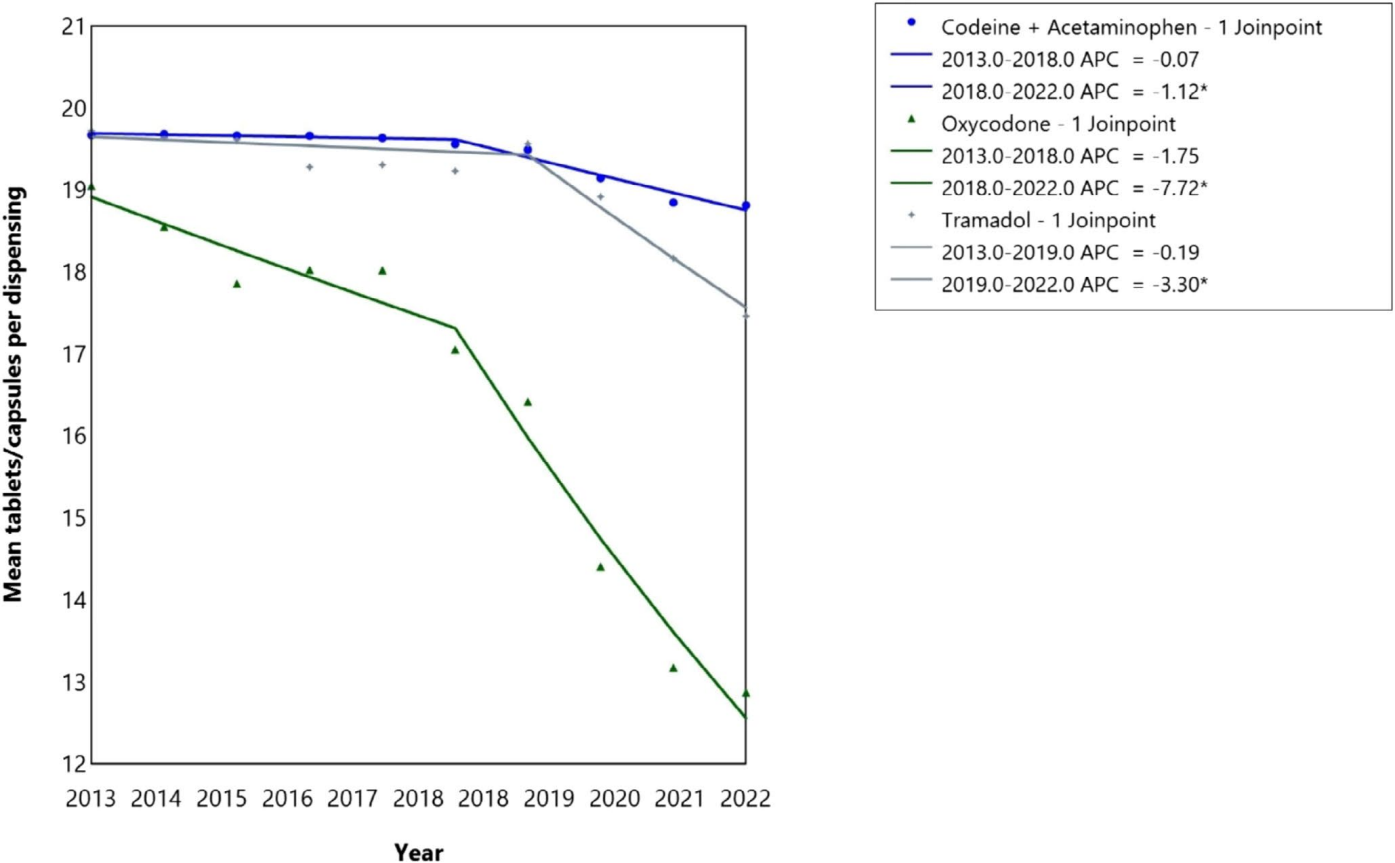
Temporal trends in the mean number of tablets/capsules dispensed per dental opioid drugs in Australia between 2013 and 2022. *Indicates that the Annual percent change (APC) is significantly different from zero at the alpha = 0.05 level.

A significant reduction in the mean quantity of opioid tablets/capsules dispensed per prescription was observed between 2018 and 2022 (Figure 2). The mean number of codeine 30 mg/acetaminophen 500 mg tablets per prescription reduced by 1.1% between 2018 and 2022 (95% CI: 0.9–1.5). The mean number of tramadol 50 mg tablets/capsules per prescription reduced by 3.3% between 2019 and 2022 (95% CI: 2.3–6.0). The largest reduction was seen in oxycodone where the mean number of tablets per prescription decreased by 7.7% between 2018 and 2022 (95% CI: 5.4–14.9).

## 4 Discussion

This first comprehensive assessment of Australian opioid dispensing from dental prescriptions demonstrates some positive changes through smaller opioid quantities being dispensed, and 99% of patients who were dispensed opioids prescribed by dentists had less than five dispensings over the 10‐year observation period. However, the overall average increase in the number of opioid prescriptions by 4.4% per year is concerning given the poor effectiveness of opioids for dental pain [7, 15]. Additionally, the small but significant dispensing of opioids, particularly codeine, for children (some under 5 years old) is concerning and warrants further investigation. Furthermore, this is the first Australian evidence that a small group of patients (*n* = 590) obtained multiple opioid prescriptions from dentists, and some on the same day. These prescribing practices provide clear opportunities for designing targeted interventions to ensure safe prescribing of these potentially high‐risk medicines, as well as areas for further research.

While dental opioid prescribing has been decreasing in the US [4], our study demonstrated a sustained increase in opioid dispensing over a decade in an Australian population. The rate of growth in oxycodone dispensing exceeded that of codeine with acetaminophen, likely due to a recent change in Australian dental guidelines, where oxycodone was recommended as the preferred opioid [6]. In the present study, most dispensing was to those aged 20–39, which may be explained by prescribing opioids for third molar extractions. However, multiple studies have demonstrated the limited effectiveness of opioids for dental pain and particularly for postsurgical third molar extraction pain [15, 16]. A randomised controlled trial demonstrated that the addition of codeine to a regimen of ibuprofen and acetaminophen did not produce any extra pain relief for third molar extractions [15]. The routine prescribing of opioids for dental procedures is also questioned, with a prospective study demonstrating that only 7% of patients prescribed an opioid after third molar surgery required oxycodone for pain relief, with the remainder finding acetaminophen and ibuprofen sufficient [17]. As such, targeted dental education on the appropriate use of opioids for third molar extractions and other dental indications may be warranted.

A positive finding from this study is the reduced quantities dispensed of opioids prescribed by Australian dentists, suggesting a trend towards a shorter duration of use. In contrast, dental prescriptions exceeding the recommended duration have been demonstrated in the US [1]. While it is common for patients to be prescribed larger quantities of opioids than what they require in dentistry [17], the advantage of reduced prescribed quantities is the smaller number of leftover pills, as this is the most common source of opioids that are misused [18]. Prescribing smaller quantities was a recommendation in the latest version of Australian national dental prescribing guidelines that was published in 2019 [6]. In June 2020, an initiative from the Australian Government to reduce the number of leftover opioid pills included implementing smaller pack sizes of several short‐acting opioids [19]. Both of these may be contributing factors to the reduced quantities of opioids prescribed by dentists in the present study.

An unexpected finding from this study was that men were 21% more likely to be prescribed an opioid by a dentist compared with women. There is no gender difference in the experience of postoperative pain after surgical third molar extractions and oral surgery [20]. Similarly, the experience of pain after endodontic treatment was similar by sex, although reported more frequently in women [21]. A study of opioid prescribing after surgical tooth extractions in the US from 2000 to 2010 showed that similar proportions of women and men received opioid prescriptions [22]. Multivariable analysis of dental visits to the emergency department from 2015 to 2017, based on a national sample from the National Hospital Ambulatory Medical Care Survey, demonstrated there was no sex difference in receiving a dental opioid prescription [23]. The reason why women are less likely to be prescribed opioids is unclear. However, the Australian National Health Survey showed that more women seek dental treatment compared to men (52% women compared to 45% men in 2020–2021) [24]. Thus, it may be that more women receive preventive dental care, warranting less acute treatment and pain relief. Nonetheless, this is another area for future research to understand the factors influencing management of dental pain in women and men.

A concerning finding from the present study was the prescriptions of codeine to children less than 12 years. Opioids are not recommended for children for postoperative dental pain by Australian or US clinical guidelines [6, 25, 26]. A systematic review of analgesics for acute pain in the paediatric population showed that compared to acetaminophen and NSAIDs, opioids had a greater risk of central nervous system effects, such as dizziness and drowsiness [27]. Furthermore, codeine is contraindicated in children less than 12 years due to the unpredictable pharmacogenetic polymorphism of the enzyme cytochrome P450 2D6, rendering some patients ‘rapid metabolisers’ [28]. Case reports have been published of deaths of children who were rapid metabolisers and were prescribed codeine after tonsillectomy, resulting in respiratory depression from increased production of morphine [29]. Given these findings, continuing education for dentists can be intentionally directed towards prescribing appropriately and highlighting the potential harms of opioids in the paediatric population.

The present study demonstrates that approximately 1% of the Australians dispensed an opioid for dental pain received multiple opioid prescriptions from dentists, and some on the same day. The misuse of pharmaceutical and illegal opioids in Australia is a major public health issue, where approximately 55,000 patients received pharmacotherapy for opioid dependence in a ‘snapshot’ day in 2022 [30]. Due to the misuse of opioids, one initiative by the Australian Therapeutic Goods Administration was to up‐schedule codeine to prescription‐only on 1 February 2019 in Australia [31]. Despite this measure, and while Australian medical opioid prescribing did not change, dental opioid prescribing of oxycodone increased immediately after the codeine up‐schedule by 24% [5, 32]. Furthermore, several studies from the US and Canada have demonstrated the association of dental opioid prescriptions with persistent opioid use and abuse [8, 33, 34]. A cross‐sectional study using commercial health insurance data showed that of the young adults who initiated opioid use with a prescription from their dentist, 6% developed an opioid abuse‐related diagnosis, compared to 0.4% of the control group [34]. This present study demonstrated that a very small cohort of patients received multiple dental opioid prescriptions, highlighting that Australian dentists may not be exempt from seeing patients potentially seeking high‐risk medicines from multiple prescribers. Investigating the details of these cases and understanding the contribution of Australian dental opioid prescribing towards persistent opioid use and misuse require further research.

This study has some limitations. Firstly, dental prescriptions dispensed to public hospital inpatients and private prescriptions were not included in the dataset. Private prescriptions are those that do not meet the eligibility for subsidisation by the PBS (e.g., not listed for subsidisation, ineligible doses/quantities), and thus are fully paid by the patient. Secondly, as the data were not linked to dental records, it is not possible to fully analyse the appropriateness of the prescriptions. The PBS dataset can have some missing data, which is typically minimal. Unfortunately, precise values cannot be provided as the amount of missing data changes based on when the data is extracted. Finally, the PBS data were not collected for research purposes, so a limited set of variables are included, restricting detailed demographic and clinical analysis.

This study highlights some clear opportunities for action for Australian dentistry. As opioid dispensing is increasing, interventions can include targeted education, audit and feedback, interdisciplinary pain management and risk‐assessment for opioid‐naïve patients [1, 35]. Integrated pharmacy services have been shown to be effective in reducing inappropriate dental opioid prescribing in the US [36]. Customising the intervention and investigating enablers and barriers to uptake is needed for the successful implementation of interventions to improve prescribing in the Australian dental setting. Given the small cohort of patients who received multiple opioid dental prescriptions, dentists should be provided with appropriate support to enable better informed prescribing decisions. All Australian states have real‐time prescription monitoring (RTPM) programs, and dentists are provided access in most states, but not all. Furthermore, not all states that provide access mandate the use of these systems. Without being mandated, dentists have been shown to have low uptake of drug monitoring programmes [37]. RTPM programs can inform prescribing choices, addiction screening or help with referral [38]. This present study demonstrates the value and importance of enforcing the mandatory use of RTPM and providing access for all Australian dentists when prescribing high‐risk medicines.

## 5 Conclusions

Australian dental opioid prescribing has increased over a decade to 2022. Quantities of opioids dispensed per prescription have reduced in recent years. However, there is some potentially concerning prescribing of opioids regarding prescribing for children and excessive/same‐day dispensing. Evidence‐based tailored opioid stewardship interventions need to include dentists, and future research can investigate the contribution of dentistry towards persistent opioid use. Guidelines should be clear about not prescribing opioids where a low likelihood of dental pain will be experienced, or for patients who can tolerate NSAIDs. Other recommended initiatives include providing access for all Australian dentists to RTPM, and mandating its use to help inform prescribing decisions.

## Author Contributions

**L.T**. made substantial contributions to the conception of the work, interpretations of the data, drafting the work and reviewing it critically for important intellectual content. **M.T**. made substantial contributions to the design of the work, the analysis and interpretation of the data, drafting the work and reviewing it critically for important intellectual content. **E.K**. made substantial contributions to the analysis and interpretation of the data for the work and drafting the work. **F.S**. made substantial contributions to the acquisition and interpretation of the data and reviewing the work critically for important intellectual content. **M.L**. made substantial contributions with the interpretation of data and reviewing the work critically for important intellectual content. **M.M.c**. made substantial contributions with the interpretation of the data and reviewing the work critically for important intellectual content. **C.E.‐B.** made substantial contributions to the acquisition and interpretation of the data and reviewing the work critically for important intellectual content. **A.P**. made substantial contributions to the interpretation of the data and reviewing the work critically for important intellectual content. **D.P**. made substantial contributions to the acquisition and interpretation of the data and reviewing the work critically for important intellectual content. **A.P**. made substantial contributions to the analysis and interpretation of the data and reviewing the work critically for important intellectual content. All authors give final approval of the version to be published and agree to be accountable for all aspects of the work in ensuring that questions related to the accuracy or integrity of any part of the work are appropriately investigated and resolved.

## Conflicts of Interest

Christopher Etherton‐Beer is the Chair of the Drug Utilisation Sub‐Committee of the Pharmaceutical Benefits Advisory Committee; the views expressed in this paper do not represent those of the Committee.

## Supporting information


**Table S1:** STROBE Statement—Checklist of items that should be included in reports of cohort studies
**Table S2:** Annual dentist opioid prescription; incidence rate per 1000 person years.
**Table S3:** The types of opioid drugs commonly dispensed by dentists in Australia between 2013 and 2022.
**Figure S1:** Temporal trends in crude incidence rate for the supply of all dentist prescriptions and opioid dentist prescriptions: Australia 2013–2022.
**Figure S2:** Temporal trends in mean MME per dentist prescription: Australia 2013–2022.

## Data Availability

The authors direct any data requests to the data custodians: Services Australia.
