# Supplementary material for: Trends of Dispensed Opioids Prescribed by Australian Dentists: 2013–2022
Source: Community Dent Oral Epidemiol. 2025 Oct 7;54(1):69–76. doi: 10.1111/cdoe.70022 (PMC12808857; doi:10.1111/cdoe.70022)
Supplement: Supplementary file 1 — Table S1: STROBE Statement—Checklist of items that should be included in reports of cohort studies Table S2: Annual dentist opioid prescription; incidence rate per 1000 person years. Table S3: The types of opioid drugs commonly dispensed by dentists in Australia between 2013 and 2022. Figure S1: Temporal trends in crude incidence rate for the supply of all dentist prescriptions and opioid dentist prescriptions: Australia 2013–2022. Figure S2: Temporal trends in mean MME per dentist prescription: Australia 2013–2022. [file CDOE-54-69-s001.docx]

**Supplementary Table 1: STROBE Statement - Checklist of items that should be included in reports of cohort studies**

|  | Item No | Recommendation | Page No |
| --- | --- | --- | --- |
| **Title and abstract** | 1 | (*a*) Indicate the study’s design with a commonly used term in the title or the abstract | 3 |
|  |  | (*b*) Provide in the abstract an informative and balanced summary of what was done and what was found | 3 |
| Introduction | | |  |
| Background/rationale | 2 | Explain the scientific background and rationale for the investigation being reported | 4 |
| Objectives | 3 | State specific objectives, including any prespecified hypotheses | 4 |
| Methods | | |  |
| Study design | 4 | Present key elements of study design early in the paper | 4 |
| Setting | 5 | Describe the setting, locations, and relevant dates, including periods of recruitment, exposure, follow-up, and data collection | 5 |
| Participants | 6 | (*a*) Give the eligibility criteria, and the sources and methods of selection of participants. Describe methods of follow-up | 4-5 |
|  |  | (*b*) For matched studies, give matching criteria and number of exposed and unexposed | N/A |
| Variables | 7 | Clearly define all outcomes, exposures, predictors, potential confounders, and effect modifiers. Give diagnostic criteria, if applicable | 5 |
| Data sources/ measurement | 8* | For each variable of interest, give sources of data and details of methods of assessment (measurement). Describe comparability of assessment methods if there is more than one group | N/A |
| Bias | 9 | Describe any efforts to address potential sources of bias | N/A |
| Study size | 10 | Explain how the study size was arrived at | 4 |
| Quantitative variables | 11 | Explain how quantitative variables were handled in the analyses. If applicable, describe which groupings were chosen and why | 5-6 |
| Statistical methods | 12 | (*a*) Describe all statistical methods, including those used to control for confounding | 5-6 |
|  |  | (*b*) Describe any methods used to examine subgroups and interactions | 5-6 |
|  |  | (*c*) Explain how missing data were addressed | 5-6 |
|  |  | (*d*) If applicable, explain how loss to follow-up was addressed | 5-6 |
|  |  | (*e*) Describe any sensitivity analyses | 5-6 |
| Results | | |  |
| Participants | 13* | (a) Report numbers of individuals at each stage of study—eg numbers potentially eligible, examined for eligibility, confirmed eligible, included in the study, completing follow-up, and analysed | 6 |
|  |  | (b) Give reasons for non-participation at each stage | N/A |
|  |  | (c) Consider use of a flow diagram | N/A |
| Descriptive data | 14* | (a) Give characteristics of study participants (eg demographic, clinical, social) and information on exposures and potential confounders | 6-7 |
|  |  | (b) Indicate number of participants with missing data for each variable of interest | N/A |
|  |  | (c) Summarise follow-up time (eg, average and total amount) | 6 |
| Outcome data | 15* | Report numbers of outcome events or summary measures over time | 6-7 |
| Main results | 16 | (*a*) Give unadjusted estimates and, if applicable, confounder-adjusted estimates and their precision (eg, 95% confidence interval). Make clear which confounders were adjusted for and why they were included | 6-7 |
|  |  | (*b*) Report category boundaries when continuous variables were categorized | 6-8 |
|  |  | (*c*) If relevant, consider translating estimates of relative risk into absolute risk for a meaningful time period | N/A |
| Other analyses | 17 | Report other analyses done—eg analyses of subgroups and interactions, and sensitivity analyses | 7-8 |
| Discussion | | |  |
| Key results | 18 | Summarise key results with reference to study objectives | 8 |
| Limitations | 19 | Discuss limitations of the study, taking into account sources of potential bias or imprecision. Discuss both direction and magnitude of any potential bias | 10-11 |
| Interpretation | 20 | Give a cautious overall interpretation of results considering objectives, limitations, multiplicity of analyses, results from similar studies, and other relevant evidence | 8-11 |
| Generalisability | 21 | Discuss the generalisability (external validity) of the study results | 11 |
| Other information | | |  |
| Funding | 22 | Give the source of funding and the role of the funders for the present study and, if applicable, for the original study on which the present article is based | 12 |

*Give information separately for exposed and unexposed groups.

**Supplementary Table 2: Annual dentist opioid prescription; incidence rate per 1,000 person years.**

| Year | Person Time  (years) | Opioid prescription | | Codeine +  Acetaminophen | | Oxycodone | | Tramadol | | Mean MME per  prescription (SD) |
| --- | --- | --- | --- | --- | --- | --- | --- | --- | --- | --- |
|  |  | n | IR (95%CI) | n | IR (95%CI) | n | IR (95%CI) | n | IR (95%CI) |  |
| 2013 | 1,533,577 | 15,147 | 9.9 (9.7, 10.0) | 14,759 | 9.6 (9.5, 9.8) | 260 | 0.17 (0.15, 0.19) | 120 | 0.08 (0.07, 0.09) | 89.7 (12.4) |
| 2014 | 1,659,117 | 15,464 | 9.3 (9.2, 9.5) | 14,995 | 9.0 (8.9, 9.2) | 306 | 0.18 (0.16, 0.21) | 148 | 0.09 0.08, 0.10) | 89.8 (11.8) |
| 2015 | 1,690,820 | 15,573 | 9.2 (9.1, 9.4) | 15,044 | 8.9 (8.8, 9.0) | 345 | 0.20 (0.18, 0.23) | 167 | 0.10 (0.08, 0.11) | 89.7 (12.3) |
| 2016 | 1,663,951 | 15,725 | 9.5 (9.3, 9.6) | 15,176 | 9.1 (9.0, 9.3) | 371 | 0.22 (0.20, 0.25) | 166 | 0.10 (0.09, 0.12) | 89.7 (12.3) |
| 2017 | 1,667,553 | 16,257 | 9.8 (9.6, 9.9) | 15,674 | 9.4 (9.3, 9.6) | 414 | 0.25 (0.23, 0.27) | 163 | 0.10 (0.08, 0.11) | 89.7 (12.7) |
| 2018 | 1,666,253 | 19,730 | 11.8 (11.7, 12.0) | 19,000 | 11.4 (11.2, 11.6) | 484 | 0.29 (0.27, 0.32) | 224 | 0.13 (0.12, 0.15) | 89.2 (12.9) |
| 2019 | 1,702,400 | 20,360 | 12.0 (11.8, 12.1) | 19,636 | 11.5 (11.4, 11.7) | 488 | 0.29 (0.26, 0.31) | 217 | 0.13 (0.11, 0.15) | 88.7 (13.0) |
| 2020 | 1,642,254 | 21,841 | 13.3 (13.1, 13.5) | 20,680 | 12.6 (12.4, 12.8) | 870 | 0.53 (0.50, 0.57) | 274 | 0.17 (0.15, 0.19) | 87.2 (16.3) |
| 2021 | 1,620,316 | 20,970 | 12.9 (12.8, 13.1) | 19,481 | 12.0 (11.9, 12.2) | 1,193 | 0.74 (0.70, 0.78) | 286 | 0.18 (0.16, 0.20) | 85.8 (17.9) |
| 2022 | 1,737,285 | 21,421 | 12.3 (12.2, 12.5) | 19,808 | 11.4 (11.2, 11.6) | 1,324 | 0.76 (0.72, 0.80) | 281 | 0.16 (0.14, 0.18) | 85.5 (18.4) |
|  | 16,583,526 | 182,448 | 11.0 (11.0, 11.1) | 174,253 | 10.5 (10.5, 10.6) | 6,055 | 0.37 (0.36, 0.37) | 2,046 | 0.12 (0.12, 0.13) | 88.3 (14.7) |

*IR: Incidence Rate per 1,000 person-years. ^Time under observation: Includes all individuals in the sample who received a PBS prescription during that calendar year. Multiple prescription for the same patient on the same day are considered a single incident.

MME: Morphine Milligram Equivalent per opioid medication dispensed. SD: Standard Deviation.

**Supplementary Table 3: The types of opioid drugs commonly dispensed by dentists in Australia between 2013 and 2022.**

| **Opioid type** | **N (%)** | **IR (95%CI)** |
| --- | --- | --- |
| Codeine phosphate 30mg + acetaminophen 500mg | 174,781 (95.4%) | 7.74 (7.70, 7.78) |
| Oxycodone hydrochloride 5mg, 10mg, 1mg/mL | 6,257 (3.5%) | 0.27 (0.26, 0.28) |
| Tramadol hydrochloride 50mg | 2,131 (1.2%) | 0.09 (0.09, 0.09) |
| Codeine phosphate 30mg | 129 (<0.1%) |  |
| Morphine sulfate 30mg | 4 (<0.1%) |  |
| Hydromorphone hydrochloride 2mg | 1 (<0.1%) |  |

## **Supplementary Figure 1: Temporal trends in crude incidence rate for the supply of all dentist prescriptions and opioid dentist prescriptions: Australia 2013-2022.**


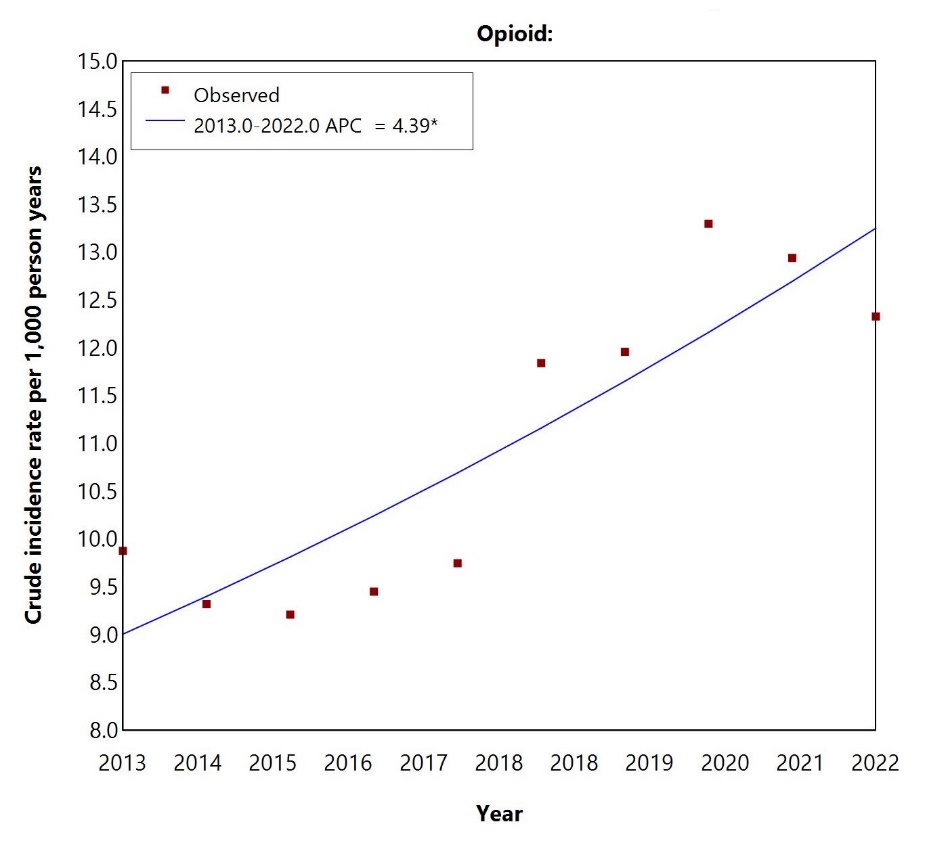

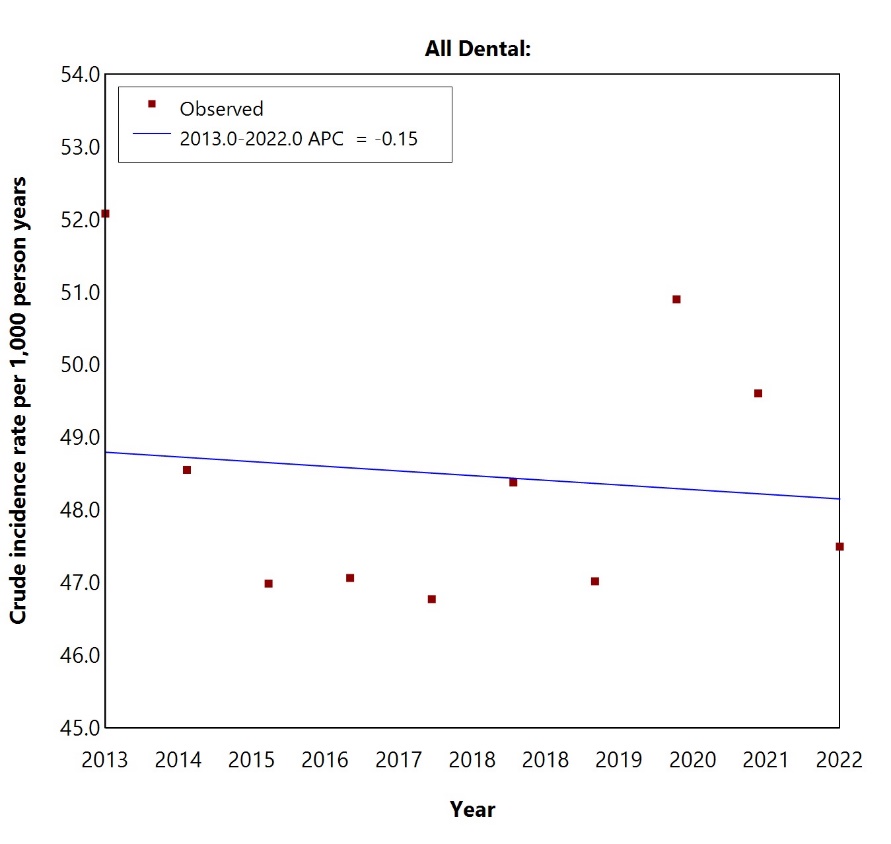


*Indicates that the Annual percent change (APC) is significantly different from zero at the alpha = 0.05 level.

## **Supplementary Figure 2: Temporal trends in mean MME per dentist prescription: Australia 2013-2022.**


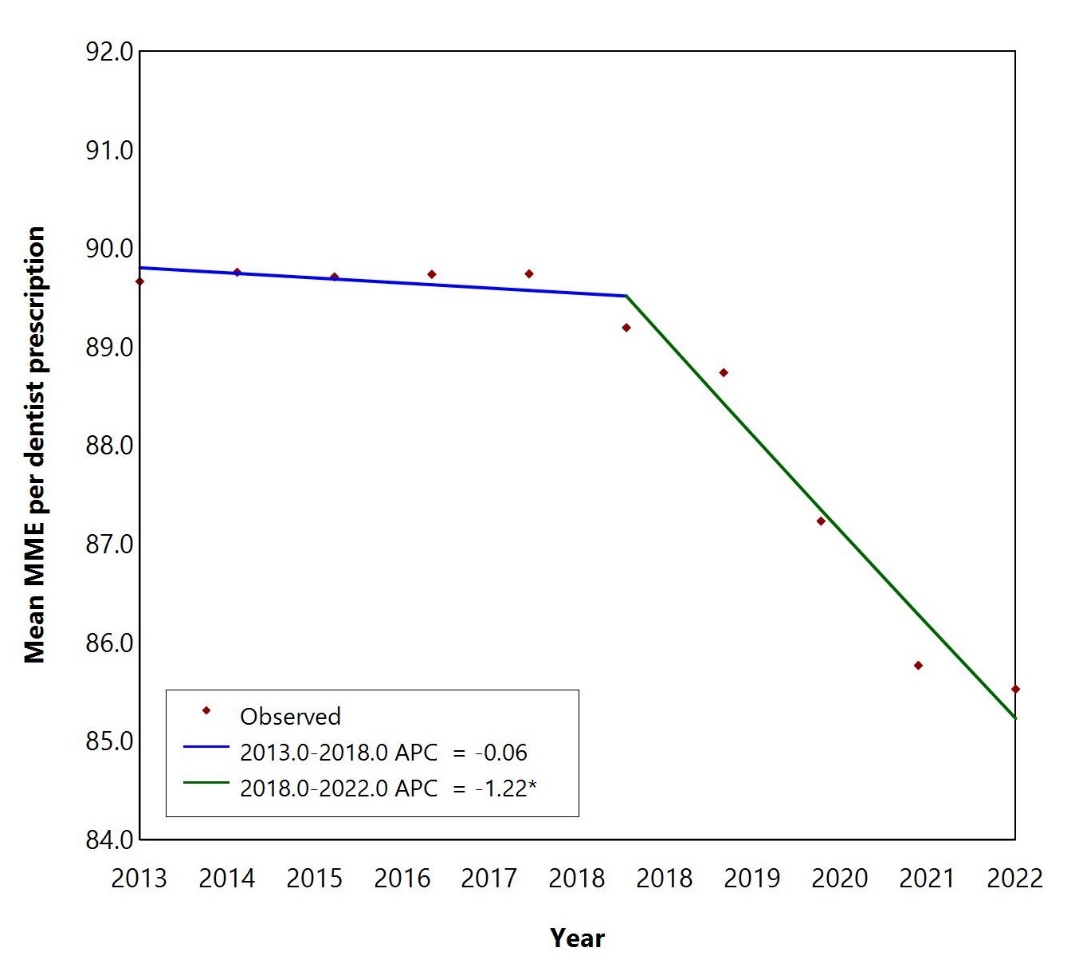


*Indicates that the Annual percent change (APC) is significantly different from zero at the alpha = 0.05 level.
